# Anti‐tumour effects of a dual cancer‐specific oncolytic adenovirus on Breast Cancer Stem cells

**DOI:** 10.1111/jcmm.16113

**Published:** 2020-12-11

**Authors:** Wenjie Li, Yiquan Li, Yingli Cui, Shanzhi Li, Yilong Zhu, Chao Shang, Gaojie Song, Zirui Liu, Zhiru Xiu, Jianan Cong, Tingyu Li, Xiao Li, Lili Sun, Ningyi Jin

**Affiliations:** ^1^ College of Animal Science and Technology Guangxi University Nanning China; ^2^ Academician Workstation of Jilin Province Changchun University of Chinese Medicine Changchun China; ^3^ Institute of Military Veterinary Medicine Academy of Military Medical Science Changchun China; ^4^ Department of Gynecologic Oncology First Hospital of Jilin University Changchun China; ^5^ Jiangsu Co‐innovation Center for Prevention and Control of Important Animal Infectious Diseases and Zoonoses Yangzhou China; ^6^ Department of Head and Neck Surgery Tumor Hospital of Jilin Province Changchun China

**Keywords:** apoptin, apoptosis, breast cancer stem cells, recombinant adenovirus

## Abstract

Apoptin can specifically kill cancer cells but has no toxicity to normal cells. Human telomerase reverse transcriptase (hTERT) can act as a tumour‐specific promoter by triggering the expression of certain genes in tumour cells. This study aims to investigate the inhibitory effects and to explore the inhibitory pathway of a dual cancer‐specific recombinant adenovirus (Ad‐apoptin‐hTERTp‐E1a, Ad‐VT) on breast cancer stem cells. Breast cancer cell spheres were obtained from MCF‐7 cells through serum‐free suspension culture. The cell spheres were detected by flow cytometry for CD44^+^ CD24^−^ cell subsets. The stemness of MCF‐7‐CSC cells was confirmed by in vivo tumorigenesis experiments. The inhibitory effect of the recombinant adenoviruses on MCF‐7‐CSC cells was evaluated by CCK‐8 assay. In addition, the stemness of adenovirus‐infected MCF‐7‐CSC cells was analysed by testing the presence of CD44^+^ CD24^−^ cell subsets. The ability of the recombinant adenovirus to induce MCF‐7‐CSC cell apoptosis was detected by staining JC‐1, TMRM and Annexin V. Our results showed that a significantly higher proportion of the CD44^+^ CD24^−^ cell subsets was present in MCF‐7‐CSC cells with a significantly increased expression of stem cell marker proteins. The MCF‐7‐CSC cells, whlist exhibited a strong tumorigenic ability with a certain degree of stemness in mice, were shown to be strongly inhibited by recombinant adenovirus Ad‐VT through cell apoptosis. In addition, Ad‐VT was shown to exert a killing effect on BCSCs. These results provide a new theoretical basis for the future treatment of breast cancer.

## 1 INTRODUCTION

Cancer, a complex disease characterized by uncontrolled cell growth and metastasis, has caused millions of fatalities annually worldwide. The development of tumours is contributed by different factors, either externally (unhealthy lifestyles and pollutants in the living environment), or internally (hormonal changes, immune response and genetics).^1^ Both internal and external factors can contribute to the onset of cancer either individually, or collectively. Tumour development can also be accompanied by additional factors such as cancer stem cells (CSCs; also known as tumour/cancer initiation cells or stem cell‐like cancer cells) that are responsible for initiating cancer. There are different tumour subtypes with different morphologies and behaviours, although the source of tumour heterogeneity remains unclear. CSCs are a specific subset of cancer cells with basic self‐renewal and differentiation properties, which can produce tumours with genomic and phenotypic heterogeneity.^2^ Accordingly, tumour heterogeneity and malignancy progression are contributed by a small number of cancer cells with stem cell‐like properties, rather than by the vast majority of cancer cells. Firstly proposed in leukaemia,^3^ CSCs have later been found to be present in solid tumour breast cancer.^4^ These stem cell‐like breast cancer cells display expression markers similar to those found in pluripotent progenitor cells, suggesting that these breast cancer stem cells (BCSCs) may be derived from mammary stem cells (MaSCs).^5^ The presence of CSCs has been reported to occur in almost all types of malignant haematological tumours and solid tumours.^5^ Like normal stem cells, CSCs are capable of self‐renewal and differentiation. These slow‐growing cells can possibly be contributed by three different factors: (1) stem cell mutations; (2) adult stem cells that produce cancer stem cells; and (3) tissue microenvironment that favours the dedifferentiation of mutant cells to become stem cells. Regardless of their stem cell characteristics, these cancer stem cells can survive, either as resting or migratory cells. Studies have shown that migratory cancer stem cells undergo epithelial mesenchymal transformation, causing cancer proliferation.^6^ The migration behaviour of cancer stem cells is one of the biggest challenges cancer treatment, because migratory cells can not only change their morphology, they are resistant therapeutic drugs. Hence, one of the potential methods for the treatment of cancer is targeted therapy, which utilizes different specific recognition markers and signal transduction pathways, separately or combined with targeted quiescent phases, drug efflux cells and apoptosis‐resistant cells.^7^ Differences in the expression profile of cancer markers can be used to facilitate the design of treatment strategies.

CSCs have been shown to promote tumour initiation and metastasis, leading to therapeutic resistance.^8^ Consequently, breast cancer with a high proportion of CSCs is associated with poor treatment outcomes. Some studies have shown that BCSCs exhibit relative resistance to conventional treatment in preclinical models and clinical trials. Consistently, at in vitro settings, paclitaxel was observed to be enriched for cells expressing BCSC phenotypes due to chemotherapy resistance.^9^ Clinical studies have shown an increased expression of CD44^+^CD24^−^ phenotype in primary tumours after chemotherapy.^10^ It has also been found that BCSCs in different breast cancer cell line models are resistant to radiation therapy.^11^ In addition, preclinical studies have demonstrated that an enhanced resistance to endocrine therapy is associated with the increased proportion of BCSCs,^12^ indicating that BCSCs have an intrinsic resistance to anticancer therapy.^13^ The inactivity nature of CSCs renders the cells to be insensitive to DNA‐damaging agents and radiation. In breast cancer, it has been shown that CSCs contribute to malignant progression, suggesting that BCSC targeting may improve therapeutic efficacy. Firstly proposed in 2003, CD44^+^CD24^−^ phenotype is by far the most commonly used marker for characterizing BCSCs.^4^ Given that BCSCs contribute to a large number of primary tumours, the combination of BCSC‐targeting agents and individualized breast cancer treatment can improve clinical outcomes.

Apoptin is a protein expressed by chicken anaemia virus that induces apoptosis in tumour cells. In normal cells, Apoptin is localized in the cytoplasm and does not cause apoptosis; whilst in tumour cells, Apoptin is localized in the nucleus. The nuclear localization of Apoptin is closely associated with the induction of tumour cell apoptosis. Apoptin is an attractive candidate for targeted tumour therapy, as it can specifically induce tumour cell apoptosis and is non‐toxic to normal cells. Meanwhile, human telomerase (hTERT) is a ribonucleoprotein that plays a role in cell senescence and immortalization, so unlimited proliferation is observed in cells with highly expressed hTERT. The promoter of hTERT appears to be inactive in most normal cells, but it is highly active in most tumour cells. The high efficiency and specificity of hTERT promoter make hTERT an effective target in tumour cells for oncolytic adenovirus.

A dual cancer‐specific oncolytic adenovirus Ad‐Apoptin‐hTERTp‐E1A (Ad‐VT) with the Apoptin and hTERT promoter was constructed together with three control viruses Ad‐Apoptin (Ad‐VP3), Ad‐hTERTp‐E1A (Ad‐T) and Ad‐Mock.^14^ The constructed cancer‐specific oncolytic adenovirus Ad‐VT can specifically replicate in tumour cells to induce cell death, because apoptin is a tumour‐specific killing protein, in addition, the hTERT promoter specifically triggers the adenovirus replication essential gene E1a. In this study, serum‐free suspension culture was used to identify CD44^+^CD24^−^ breast cancer stem cells with high tumorigenic ability through enhanced expression of tumour stem cell markers, such as ALDH1A1, C‐Myc, OCT4, NANOG, KLF 4 and SOX2. The specific ability of Ad‐VT in killing breast cancer stem cells was studied by CCK‐8 assay and detection of the presence of CD44^+^CD24^−^ cell subgroup. The ability of recombinant adenovirus in killing breast cancer stem cells was detected by the staining of JC‐1, TMRM and Annexin. Finally, the potential of recombinant adenovirus Ad‐VT in clinical application of breast cancer treatment was evaluated.

## 2 MATERIALS AND METHODS

### 2.1 Cells, viruses and animals

MCF‐7 human breast cancer cells were purchased from the Shanghai Institute of Biology Cell Bank (Shanghai, China). The cells were maintained in RPMI 1640 medium supplemented with 10% foetal bovine serum (FBS), 50 U/mL penicillin and 50 U/ml streptomycin at 37°C in an atmosphere containing 5% CO_2_. All reagents for cell culture were purchased from HyClone; GE Healthcare Life Sciences (Logan, UT, USA).

Recombinant adenoviruses Ad‐VT, Ad‐T, Ad‐VP3 and Ad‐Mock were constructed and preserved in our laboratory (Laboratory of Molecular Virology and Immunology, Institute of Military Veterinary Medicine, Academy of Military Medical Science, Changchun, China).^14^

Female NOD/SCID mice aged 4 to 5 weeks were purchased from the Experimental Animal Center of the Academy of Military Medical Sciences of China. Animal experimental protocols were approved by the Institutional Animal Care and Use Committee (IACUC) of the Chinese Academy of Military Medical Science, Changchun, China (10ZDGG007). All surgery procedures were performed under sodium pentobarbital anaesthesia, and efforts were made to minimize suffering.

### 2.2 Isolation and culture of breast cancer stem cells

MCF‐7 cells were digested and centrifuged (300 ×*g*, 5 minutes) before suspension in serum‐free medium (SFM, ie 20 ng/mL bFGF, 20 ng/mL EGF added to DME/F12, 2% B27, 1% N‐2) followed by inoculation in ultra‐low adhesion 6‐well plates at a density of 2500 cells/mL for culturing at 37°C with 5% CO_2_. The process of cell pellet formation was observed, and the culture medium was changed at an appropriate time.

After culturing for 7‐14 days, the cell pellets were collected by centrifugation before resuspension by Accutase and then digested at 37°C with 5% CO_2_ for 10 minutes. After digestion, the cells were centrifuged at 300 ×*g* for 10 min before resuspension in SFM and followed by inoculation in ultra‐low adhesion 6‐well plates at a density of 2,500 cells/ml for culturing at 37°C with 5% CO_2_.

### 2.3 Identification of CD44^+^CD24^−^ of tumour stem cells by flow cytometry

Breast cancer stem cells (MCF‐7‐CSC) were centrifuged (300 ×*g*, 5 minutes) and washed twice with PBS before digestion with 2 mL of Accutase at 37°C for 10 minutes, followed by addition of 2 mL of SFM. The cells were then counted after filtration through a 200‐mesh sterile nylon mesh before being transferred into 6 sterile 1.5 mL centrifuge tubes (4 × 10^5^ cells/tube). An additional 3 tubes of McF‐7 cells were set as controls.

Fluorescent antibody was added in the dark. The first tube of MCF‐7‐CSC without antibody treatment was used as a negative control. The cells in tube 2 and tube 3 contained only 10 µL of CD44‐FITC and 10 µL of CD24‐PE, respectively. The cells in tube 4, 5 and 6 each contained 10 µL of CD44‐FITC and 10 µL of CD24‐PE. The remaining 3 tubes of MCF‐7 cells were simultaneously treated with 10 µL of CD44‐FITC and 10 µL of CD24‐PE. The cells were then incubated at 4°C for 30 minutes in the dark before centrifugation at 300 ×*g* for 10 minutes, followed by a cleaning step twice with 1 mL of fluorescent lotion, and a fixing step with 500 µL of fluorescent fixative (PBS containing 4% formaldehyde). The cells were suspended and then transferred to a flow tube for detection.

### 2.4 Detection of the expression of tumour stem cells markers by Western blot

The marker proteins of tumour stem cells were detected by immunoblotting. MCF‐7‐CSC and MCF‐7 cells were trypsinized and collected by centrifugation at 1875 ×*g* for 5 minutes. The cell pellets were resuspended in lysis buffer, and the protein solution was collected by centrifugation at 10800 ×*g* for 5 minutes. All samples were analysed by Western blot.^15^

### 2.5 In vivo tumour formation of MCF‐7‐CSC

NOD/SCID mice were randomly divided into 9 groups. The mice in each group were subcutaneously injected with 2 mg/mL of oestradiol benzoate at a weight ratio of 1 mg/kg, once every 5 days at the back 3 days prior to cell inoculation in order to establish a tumour‐bearing model. After disinfection using alcohol cotton balls, 100 µL of cell suspension was injected into each area on the upper right, lower right, upper left and lower left of the mouse abdomen. The cell types and cell numbers of each group were as follows: Group 1: MCF‐7‐CSC at 1 × 10^2^ cells/mL; Group 2: MCF‐7‐CSC at 2 × 10^2^ cells/mL; Group 3: MCF‐7‐CSC at 5 × 10^2^ cells/mL; Group 4: MCF‐7‐CSC at 1 × 10^3^ cells/mL; Group 5: MCF‐7 cells at 1 × 10^4^ cells/mL; Group 6: MCF‐7 cells at 2 × 10^4^ cells/mL; Group 7: MCF‐7 cells at 5 × 10^4^ cells/mL; Group 8: MCF‐7 cells at 1 × 10^5^cells/mL; and Group 9: negative control.

Tumour formation was observed within 60 days of cell inoculation in the mice. The largest and the shortest tumour diameter, as well as the tumour volume, was calculated using the following formula: 0.52 × (smallest diameter)^2^ × (largest diameter).^14^, ^16^, ^17^ The tumour growth trend in mice was analysed. The tumour volume doubling time was calculated using the formula: TVDT = t×[lg*V_t_*–lg*V*_0_)], where *V*_0_ is the previous tumour volume, and *V_t_* is the next tumour volume and *t* is the time difference between two volume measurements.

### 2.6 CCK‐8 assay

MCF‐7‐CSC cell suspensions were adjusted to a density of 1 × 10^5^ cells/mL before cell SAP was separately added to a 96‐well ultra‐low adhesion culture plate at 100 μL/well (six replicate and control wells) and then cultured at 37°C with 5% CO_2_ for 24 hours. Then, three recombinant adenoviruses Ad‐VT, Ad‐T, Ad‐VP3 and Ad‐Mock were subsequently inoculated at 100 MOI, 10 MOI and 1 MOI, respectively. At 24, 48, 72 and 96 hours, the cells were added with 10 µL of CCK‐8 in the dark, and then incubated for 1‐4 hours at 37°C with 5% CO_2_ before absorbance measurement at 450 nm using a microplate reader. Cell inhibition rate was calculated using the following formula: cell inhibition rate = [(Ac–As)/(Ac–Ab)] × 100%, where As is the experimental well containing cells and recombinant adenovirus with CCK‐8 added; Ac is the control well containing cells but no recombinant adenovirus with CCK‐8 added; Ab is the blank well with only CCK‐8 but without cells and recombinant adenovirus.

### 2.7 Evaluation of the effect of recombinant adenovirus on the CD44^+^CD24^−^cell subsets of MCF‐7‐CSC by flow cytometry

MCF‐7‐CSC suspensions were adjusted to a density of 1 × 10^5^ cells/ml before cell SAP was separately added to a 6‐well ultra‐low adhesion culture plate at 2 mL/well, and cultured at 37°C with 5% CO_2_ for 24 hours. Ad‐VT was subsequently inoculated at 100 MOI. The proportion of CD44^+^CD24^−^ cell subsets was detected by flow cytometry at 48 hours.

### 2.8 Annexin V analysis

MCF‐7‐CSC suspensions were adjusted to a density of 1 × 10^5^ cells/mL before cell SAP was separately added to a 6‐well ultra‐low adhesion culture plate at 2 mL/well, and cultured at 37°C with 5% CO_2_ for 24 hours. Then, Ad‐VT, Ad‐T, Ad‐VP3 and Ad‐Mock were subsequently inoculated at 100 MOI. At 24, 48 and 72 hours, the cells were collected by centrifugation at 2,000r/min for 5min before resuspension with 500 μL of 1 × Binding Buffer, followed by addition of 5 μL of FITC and 5 μL of PI. The cells were stained for 15‐20 minutes in the dark, and then, 10 μL of cell suspension was absorbed and placed on a covered slide to be observed and photographed by confocal microscopy and fluorescence microscopy.

### 2.9 JC‐1 staining assay

JC‐1 is used to detect the qualitative and quantitative changes of mitochondrial membrane potential (MMP). MCF‐7‐CSC suspensions were adjusted to a density of 1 × 10^5^ cells/mL before cell SAP was separately added to a 6‐well ultra‐low adhesion culture plate at 2 mL/well and cultured at 37°C with 5% CO_2_ for 24 hours. Ad‐VT, Ad‐T, Ad‐VP3 and Ad‐Mock were subsequently inoculated at 100 MOI. At 48 hours, the culture solution was discarded before the cells were treated with 1 mL of JC‐1 dye diluted at 1:1000 (1 μL JC‐1 and 1 mL culture solution), followed by 15 minutes of incubation in the dark, and then, 10 μL of sample was absorbed and placed on a covered slide to be observed and photographed by confocal microscopy and fluorescence microscopy.

### 2.10 TMRM staining assay

MCF‐7‐CSC suspensions were adjusted to a density of 1 × 10^5^ cells/ml before cell SAP was separately added to a 6‐well ultra‐low adhesion culture plate at 2 mL/well and cultured at 37°C with 5% CO_2_ for 24 hours. Ad‐VT, Ad‐T, Ad‐VP3 and Ad‐Mock were subsequently inoculated at 100 MOI. At 48 hours, the culture solution was discarded before the cells were treated with 1 mL TMRM dye at 10 μg/mL, followed by 15 minutes of incubation in the dark. The cell slide was placed upside down to be observed by fluorescence microscopy. After the above treatment, the cell samples were transferred into flow tubes for and analysis using flow cytometry.

### 2.11 Statistical analyses

Statistical analyses were conducted using the data from at least three independent experiments using SPSS 20.0. *P* < 0.05 was used to indicate statistical significance. Data are presented as mean ± standard deviation (SD).

## 3 RESULTS

### 3.1 Isolation, culture and identification of MCF‐7‐CSC

**FIGURE 1:**
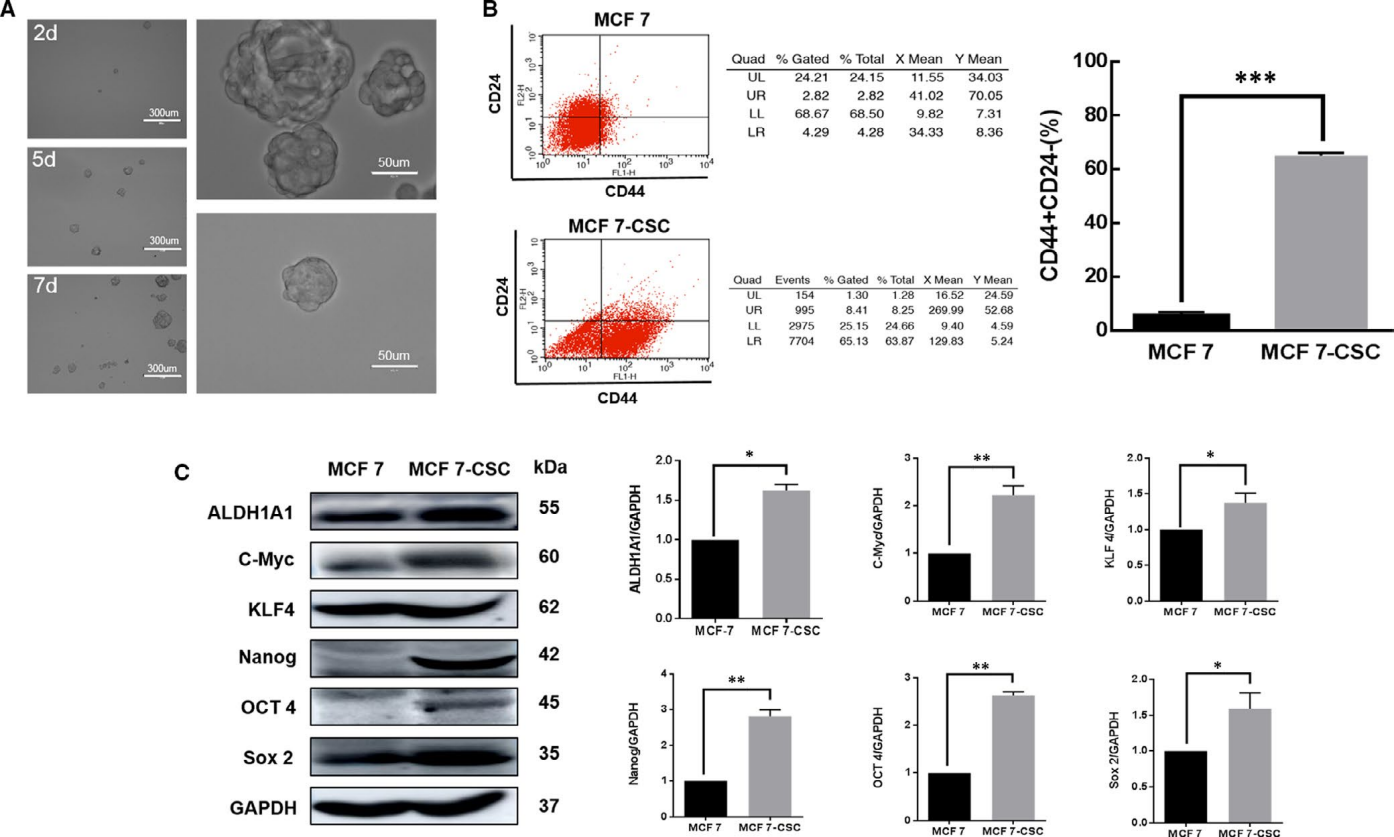
Isolation and identification of MCF‐7‐CSC. (A) A cell cluster in suspension growth formed (200×) after a 10‐day culture of MCF‐7 in serum‐free suspension. (B) Detection of CD44^+^ CD24^−^ cell subsets using flow cytometry. After serum‐free suspension culture, the proportion of CD44^+^ CD24^−^ cell subsets in MCF‐7‐CSC was significantly higher than that of MCF‐7. (C) The expression of ALDH1A1, C‐Myc, OCT4, NANOG, KLF 4 and SOX2 in MCF‐7 and MCF‐7‐CSC cells by Western blot analysis. The protein levels inMCF‐7‐CSC cells were higher than those in MCF‐7 cells. Data are presented as means ± SD (*P < 0.05 and ***P < 0.001)

A cell cluster with proliferative ability was formed in the serum‐free suspension culture of MCF‐7. The volume of cell sphere was observed to have increased with prolonged culture time (Figure 1A). The proportion of CD44^+^CD24^−^ cell subsets in the serum‐free suspension culture of MCF‐7‐CSC was significantly higher than that of MCF‐7 (*P* < 0.05) (Figure 1B), indicating that the presence of surface markers of breast cancer stem cells in MCF‐7‐CSC had increased significantly than that of in MCF‐7.

The expression levels of cancer stem cell marker proteins ALDH1A1, C‐Myc, OCT4, NANOG, KLF 4 and SOX2 were found to be significantly higher in MCF‐7‐CSC than those in MCF‐7 (Figure 1C). Taken together, the results indicate that breast cancer stem cells MCF‐7‐CSC were successfully cultured.

### 3.2 Characterization of MCF‐7‐CSC by tumour‐forming experiments in vivo

**FIGURE 2:**
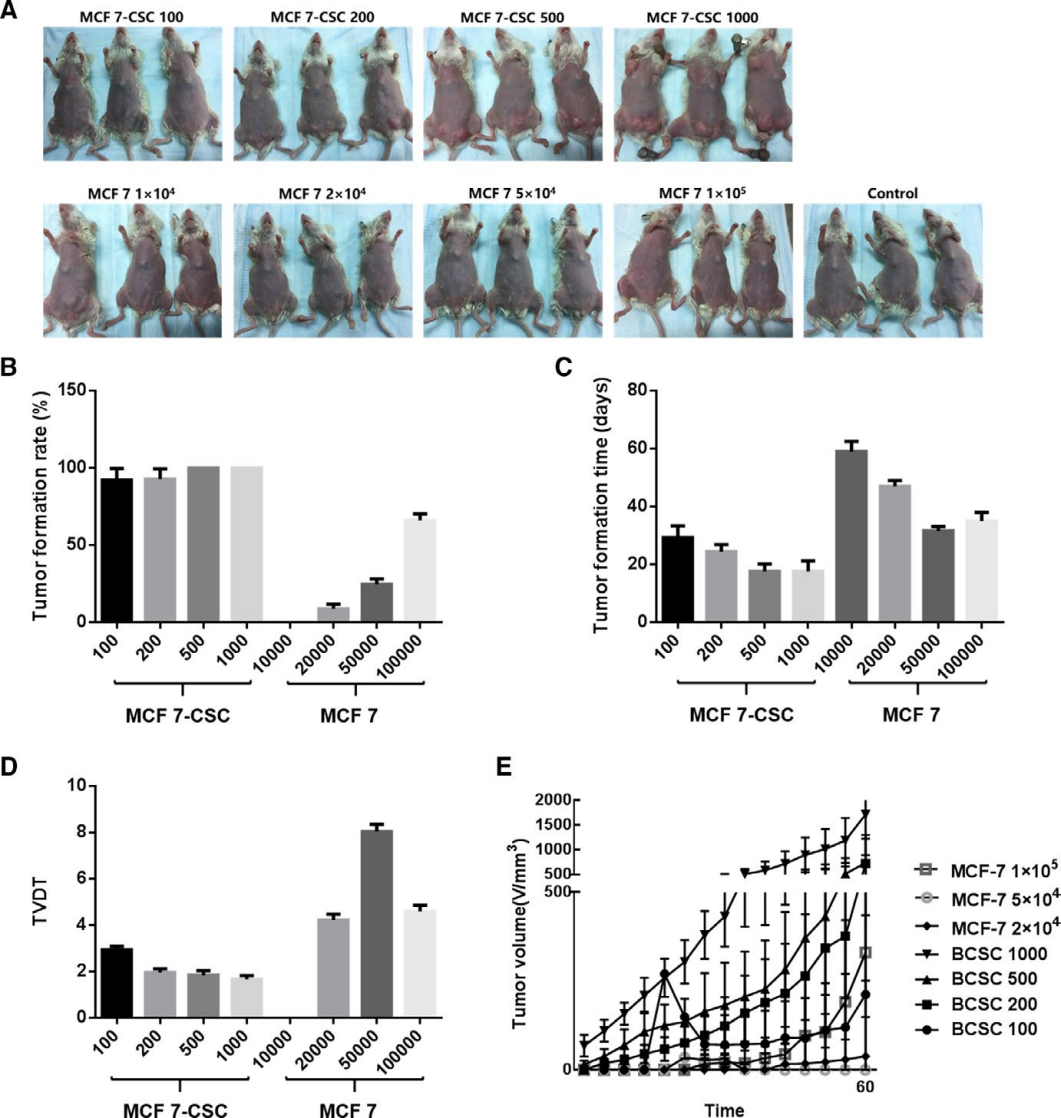
Analysis of MCF‐7‐CSC tumour formation in vivo. (A) Tumour formation in mice. As low as 100 MCF‐7‐CSC cells could induce tumour formation, whilst 1 × 10^4^ MCF‐7 cells could not induce tumour formation. (B‐D) The tumour formation rate of MCF‐7‐CSC was significantly higher than that of MCF‐7. (E) After inoculation of cells, mice in the MCF‐7‐CSC group formed tumours and the tumour volume increased rapidly, whilst mice in the MCF‐7 group formed fewer tumours, and the tumour volume growth trend of mice was slower than that of mice in the MCF‐7‐CSC group. Data are presented as mean ± SD

Mice inoculated with only 100 cells of MCF‐7‐CSC were seen to have developed tumours after only 30 days. Conversely, tumours were absent in mice inoculated with MCF‐7 even at a 100‐fold increased cell number of 1 × 10^4^. The tumour formation rate in mice with 500 MCF‐7‐CSC cells was 100%, whilst the tumour formation rate in mice with 1 × 10^5^ MCF‐7 cells was only 66.7% (Figure 2A‐D). The results indicate that MCF‐7‐CSC in serum‐free suspension culture had a stronger tumorigenic ability with certain stem cell characteristics.

After cell inoculation, whilst mice in the MCF‐7‐CSC group formed rapid‐growing tumours, mice in the MCF‐7 group formed fewer slow‐growing tumours (Figure 2E). The tumour volume doubling time of mice in the MCF‐7‐CSC group was significantly less than that of mice in the MCF‐7 group (*P* < 0.05). The tumour multiplication time of mice inoculated with MCF‐7‐CSC showed a decreasing trend with the increase of inoculated cell number (Figure 2E). Consistently, the results show that MCF‐7‐CSC with certain stem cell characteristics were successfully cultured.

### 3.3 Inhibitory effect of recombinant adenovirus on MCF‐7‐CSC

**FIGURE 3:**
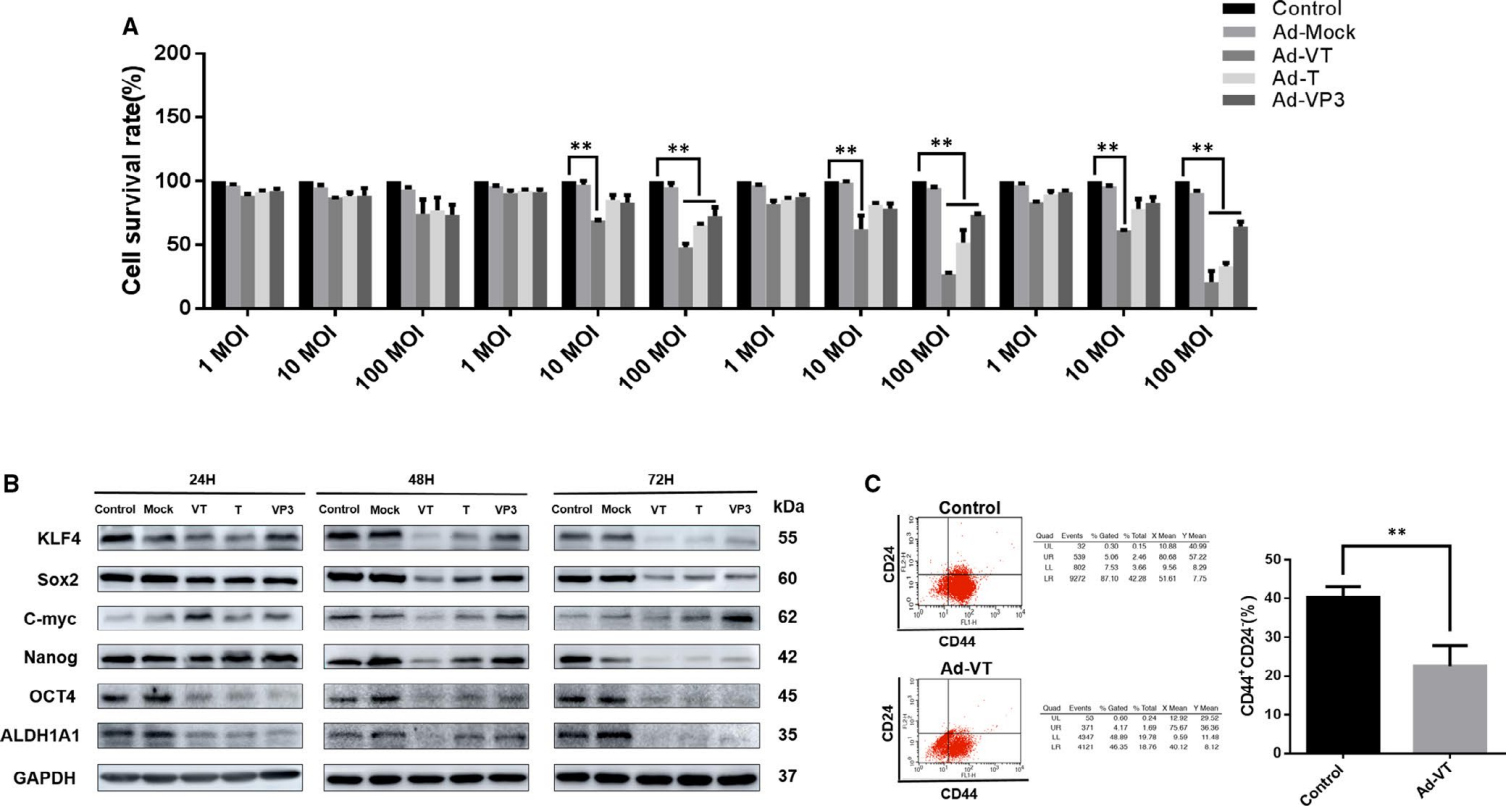
The killing and stemness effects of Ad‐VT on MCF‐7‐CSC cells. (A) After infection, the killing effects of Ad‐VT, Ad‐T, Ad‐VP3 and Ad‐Mock on MCF‐7‐CSC cells were analysed at 24, 48, 72 and 96 h. The killing effects of adenovirus on MCF‐7‐CSC cells were time‐ and dose‐dependent. The killing effects of Ad‐VT, Ad‐T and Ad‐VP3 on MCF‐7‐CSC showed the highest rates at 96 h (82%, 69% and 30%, respectively). (B) After the MCF‐7‐CSC cells were infected with Ad‐VT, the changes of CD44^+^CD24^−^ cell subsets were detected by flow cytometry. Ad‐VT could reduce the CD44^+^CD24^−^ cell subpopulation in MCF‐7‐CSC. (C) Western blot analysis of ALDH1A1, C‐Myc, OCT4, NANOG, KLF 4 and SOX2 protein expression in MCF‐7‐CSC cells infected with Ad‐VT, Ad‐T, Ad‐VP3 and Ad‐Mock. The protein levels in MCF‐7‐CSC cells were higher than those in MCF‐7 cells infected with recombinant adenoviruses. Data are means ± SD (**P < 0.01) when compared with controls

To investigate the effect of recombinant adenovirus on MCF‐7‐CSC, the cells were individually inoculated with Ad‐VT, Ad‐T, Ad‐VP3 and Ad‐Mock before CCK‐8 was added at 24, 48, 72 and 96 hours. As shown in Figure 3A, whilst the inhibitory effects of Ad‐VT and Ad‐T on MCF‐7‐CSC increased in a time‐dependent manner throughout the experimental period at the same dosage, the inhibitory effects of Ad‐VT and Ad‐T on MCF‐7‐CSC increased with a higher dosage in a dose‐dependent manner. The inhibitory effects of Ad‐VT, Ad‐T and Ad‐VP3 on MCF‐7‐CSC were significantly stronger than those of Ad‐Mock and control (*P* < 0.05). The inhibitory effects of Ad‐VT, Ad‐T and Ad‐VP3 on MCF‐7‐CSC at 96 hours showed the highest inhibition rates of 82%, 69% and 30%, respectively. Overall, Ad‐VT exhibited the highest inhibitory effects, followed by Ad‐T and Ad‐VP3, and there is no significant difference between Ad‐Mock and control group(*P* > 0.05).

### 3.4 Effect of recombinant adenovirus on the stemness of MCF‐7‐CSC

MCF‐7‐CSC inoculated with Ad‐VT showed a significantly decreased proportion of CD44^+^CD24^−^ cell subpopulation in a dose‐ and time‐dependent manner, which was significantly different from that of the control group (*P* < 0.05) (Figure 3B).

ALDH1A1, C‐myc, KLF4, Nanog, OCT4 and Sox2 that participate in the maintenance and regulation of CSC functions are used as the markers of CSC. Compared with the control group, the expression levels of CSC markers decreased in adenovirus‐infected MCF‐7‐CSC cells, especially cells infected with Ad‐VT. The expression levels of CSC markers gradually decreased after infection in a time‐dependent manner, therefore suppressing the stem cell function and decreasing the stemness of MCF‐7‐CSC cells (Figure 3C). Taken together, the results show that Ad‐VT can effectively inhibit the stemness of MCF‐7‐CSC cells.

### 3.5 Recombinant adenovirus induces cell apoptosis of MCF‐7‐CSC

**FIGURE 4:**
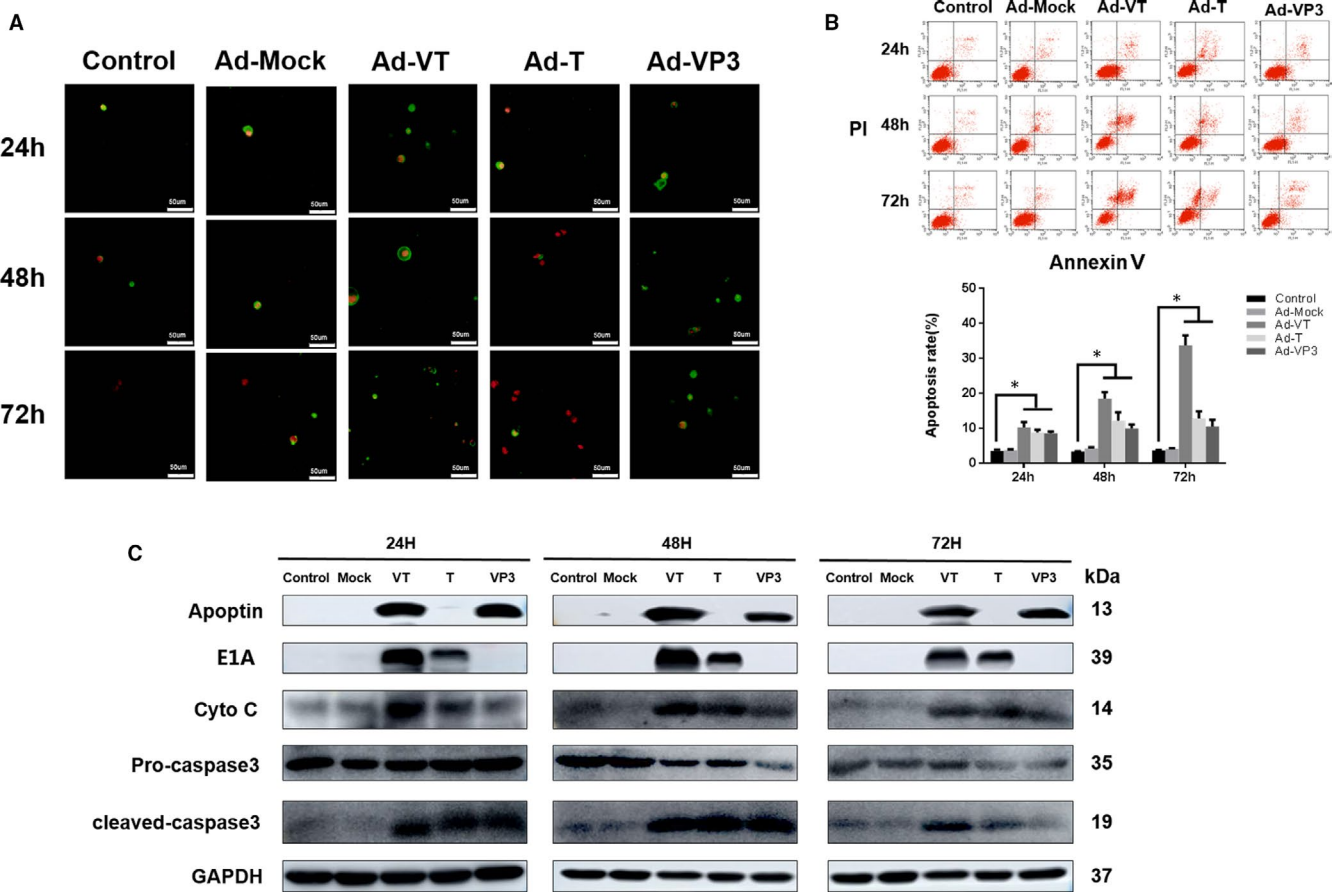
Characterization of cell death pathway induced by Ad‐VT on MCF‐7‐CSC cells. (A) Cell morphological changes were visualized by fluorescence microscopy after Annexin V FITC/PI staining. MCF‐7‐CSC cells infected with Ad‐VT, Ad‐T, Ad‐VP3 and Ad‐Mock at 24, 48 and 72 h were stained with Annexin V FITC/PI stain. Cell membrane phospholipid eversion and nuclear fragmentation of Ad‐apoptin group increased significantly over time. (B) MCF‐7‐CSC cell apoptosis was analysed by flow cytometry after Annexin V FITC/PI staining. The apoptosis level of MCF‐7‐CSC cells infected with Ad‐VT, Ad‐T and Ad‐VP3 was significantly higher than that of Ad‐Mock and control groups. Amongst them, the effect of Ad‐VT was the most significant. (C) Western blot analysis of apoptin, E1A and caspase‐3 in MCF‐7‐CSC cell extracts. Both Ad‐VT and Ad‐T could effectively express E1A protein, and Ad‐VT and Ad‐VP3 could effectively express apoptin protein. After infection with Ad‐VT, Ad‐T and Ad‐VP3, the level of cleaved‐caspase‐3 increased overtime. Amongst them, the effect of Ad‐VT was the most significant. The scale bar equals 50 μm. Data are shown as mean ± SD (*P < 0.05, **P < 0.01 and ***P < 0.001) when compared with controls

In order to detect the cell apoptosis of MCF‐7‐CSC following infection with recombinant adenovirus, Annexin V staining was firstly performed. Confocal microscope images (Figure 4A) showed that cells infected by recombinant adenovirus exhibited apoptosis features, such as cell membrane phospholipid eversion and nuclear fragmentation. Consistently, the apoptotic features were more apparent under fluorescence microscope, especially those induced by Ad‐VT.

Annexin V flow assay was performed to further analyse the apoptotic effects of recombinant adenovirus on MCF‐7‐CSC. It was found that Ad‐VT, Ad‐T and Ad‐VP3 could induce the cell apoptosis of MCF‐7‐CSC at 24, 48 and 72 hours, with Ad‐VT showing the most significant effects (Figure 4B). The apoptotic effects became stronger as the time progresses. At 72 hours, the apoptosis rates of MCF‐7‐CSC induced by Ad‐VT, Ad‐T and Ad‐VP3 reached 42.84%, 23.08% and 16.17%, respectively. Overall, the results indicate that Ad‐VT, Ad‐T and Ad‐VP3 could induce the cell apoptosis of MCF‐7‐CSC, with Ad‐VT showing the most significant effects, and there is no significant difference between Ad‐Mock and control group (*P* > 0.05).

Subsequently, the expression of apoptosis‐executing protein caspase‐3 was investigated. Our results showed that the level of cleaved‐caspase‐3 increased with time after infection with Ad‐VT, Ad‐T and Ad‐VP3. Again, this indicated that Ad‐VT, Ad‐T and Ad‐VP3 can effectively induce the cell apoptosis of MCF‐7‐CSC.

### 3.6 Recombinant adenovirus induces changes of mitochondrial membrane potential in MCF‐7‐CSC

**FIGURE 5:**
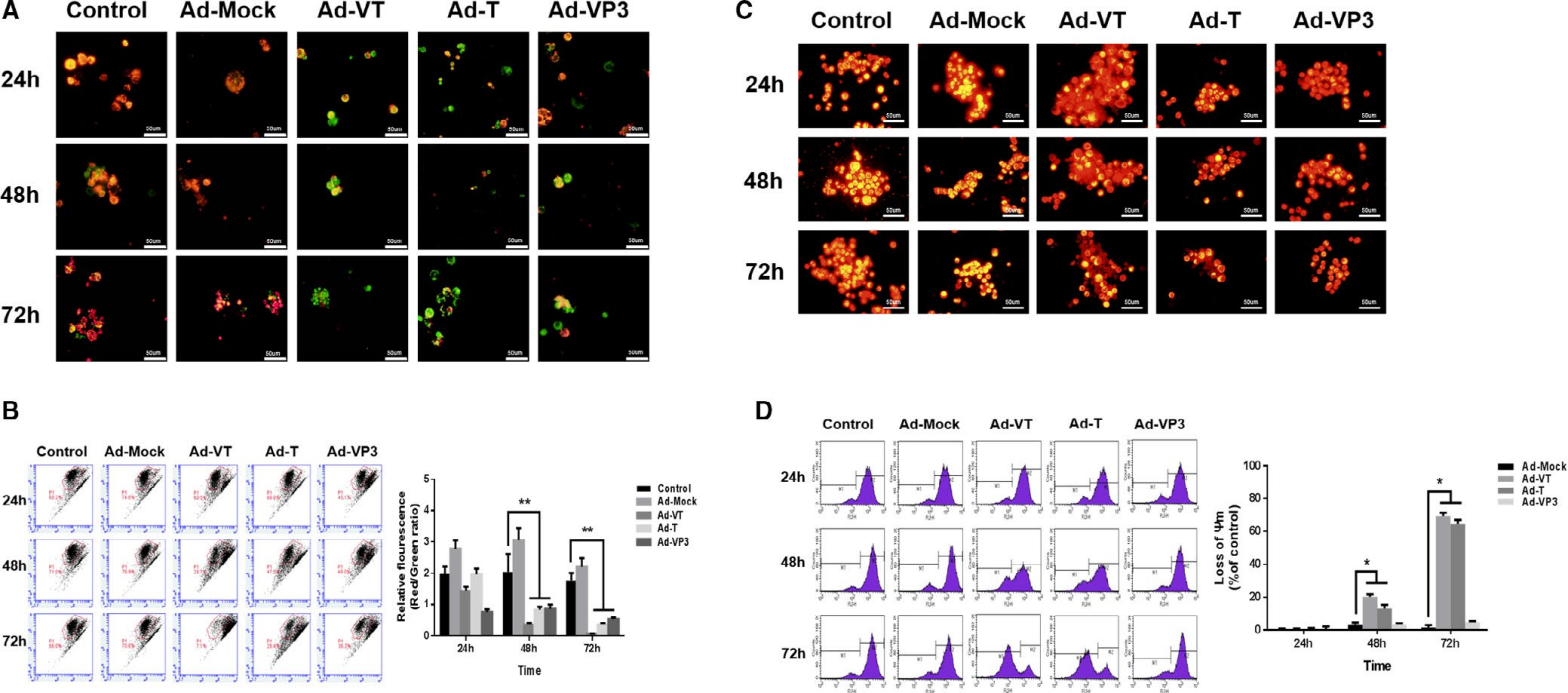
Effect of Ad‐VT on the mitochondrial membrane potential of MCF‐7‐CSC cells. (A‐B) Changes in red and green fluorescence signals measured by fluorescence microscopy after JC‐1 staining. Increased apoptosis resulted in a decrease in the ratio of red to green fluorescence. Quantitative measurement of changes in the ratio of red to green fluorescence after JC‐1 staining. Ad‐VT clearly altered the mitochondrial membrane potential (MMP). Ad‐VT had the strongest ability to induce apoptosis by affecting the MMP. (C‐D) The MMP of MCF‐7‐CSC cells was analysed by fluorescence microscopy and flow cytometry after TMRM staining. The MMP of MCF‐7‐CSC cells was significantly decreased after infection with Ad‐VT. The scale bar equals 50 μm. Data are shown as mean ± SD (*P < 0.05, **P < 0.01 and ***P < 0.001) when compared with control

To study the changes of mitochondrial membrane potential (MMP) in MCF‐7‐CSC, the cells infected with Ad‐VT, Ad‐T, Ad‐VP3 and Ad‐Mock, respectively, were stained with JC‐1 staining solution at 24, 48 and 72 hours (Figure 5A‐B). The results showed that the cell apoptosis of MCF‐7‐CSC infected by the recombinant adenoviruses was manifested by the changes of MMP across all the time points tested. The ability of Ad‐VT, Ad‐T and Ad‐VP3 in inducing the cell apoptosis of MCF‐7‐CSC was found to be increased with time. JC‐1 changed from red aggregates to green monomer in a time‐dependent manner as the number of apoptotic cells increased. Hence, there was a significant decrease (*P* < 0.05) of the red fluorescence/green fluorescence ratio as the time progressed. At 72 hours, the ratio of red fluorescence to green fluorescence was the lowest for Ad‐VT, followed by Ad‐T and Ad‐VP3, and there is no significant difference between Ad‐Mock and control group(*P* > 0.05). These results indicate that Ad‐VT, compared to the other recombinant adenovirus tested, exhibited the strongest ability to induce apoptosis by fluctuating the MMP.

Next, to analyse the qualitative and quantitative changes of MMP in MCF‐7‐CSC, TMRM staining was used (Figure 5C‐D). Whilst the mitochondrial structure was intact in the control as indicated by the strong orange fluorescence signal, mitochondrial fragmentation or a decrease in MMP took place in the adenovirus groups as the intensity of the orange fluorescence was observed to be decreased. In agreement with our results above, Ad‐VT exhibited the strongest effect in decreasing the MMP in MCF‐7‐CSC, followed by Ad‐T and Ad‐VP3, and there is no significant difference between Ad‐Mock and control group (*P* > 0.05). In summary, the recombinant adenoviruses tested had a strong apoptosis‐inducing effect on MCF‐7‐CSC cells, mainly through the mitochondrial apoptotic pathway.

## 4 DISCUSSION

The hypothesis of CSCs provides an important model for cancer research.^2^ The key role of BCSCs in breast cancer initiation, metastasis and resistance highlights the urgent need for the development of new therapeutics. An increasing number of stem cell markers that have been discovered in recent years can potentially be used to identify BCSCs.

CSCs can proliferate and form cell spheres in suspension under a serum‐free environment. In this study, the serum‐free suspension culture method enriched the BCSC in MCF‐7. The cells cultured in serum‐free suspension could form regularly shaped cell spheres after 24 hours. The cell spheres gradually became larger over time in 48 hours. A tumour cell mass with proliferative ability and with the capability of growing in suspension under serum‐free conditions was successfully obtained.

CD44 is a cell surface adhesion molecule that mediates cell‐cell and cell‐extracellular matrix (ECM) interactions by binding to hyaluronic acid (HA), whilst CD24 is a small sugar involved in the negative regulation of chemokine receptor CXCR4 protein activity in mediating breast cancer metastasis.^4^, ^10^, ^18^, ^19^ Both CD44 and CD24 have been shown as the characteristic surface markers of BCSC.^4^ BCSC is characterized by increased expression of CD44 and decreased expression of CD24.^20^, ^21^, ^22^ Therefore, BCSC at the initial stage of serum‐free suspension culture should exhibit a significantly higher ratio of CD44^+^CD24^−^ than that in MCF‐7.

Flow cytometry showed that the proportion of CD44^+^CD24^−^ cell subpopulations in BCSC increased significantly. Whilst the CD44^+^CD24^−^ cells in MCF‐7 were only 5.66%, the proportion of CD44^+^CD24^−^ cells in the cultured BCSC reached 65.07%, indicating that serum‐free suspension culture effectively enriched the CD44^+^CD24^−^ cells.

ALDH1A1, C‐Myc, OCT4, NANOG, KLF 4 and SOX2 are stem cell regulatory factors which play a crucial role in maintaining the cell's self‐renewal, proliferation and multi‐directional differentiation potential.^23^, ^24^, ^25^, ^26^, ^27^ Hence, the expression of ALDH1A1, C‐Myc, OCT4, NANOG, KLF 4 and SOX2, which are closely related to the differentiation status of stem cells, can be used as the markers of CSC. For instance, the stemness of cells can be reflected by studying the expression levels of OCT4, NANOG and KLF 4.

In this study, Western blot was used to detect the expression of ALDH1A1, C‐Myc, OCT4, NANOG, KLF 4 and SOX2 in BCSC. Our results showed that the expression levels of ALDH1A1, C‐Myc, OCT4, NANOG, KLF 4 and SOX2 were significantly higher in MCF‐7‐CSC than those in MCF‐7, indicating that MCF‐7‐CSC has stem cell characteristics.

Tumour formation experiment has been regarded as the gold standard for identifying CSCs. NOD/ SCID mice lacking the innate immune system, T lymphocytes and B lymphocytes are less susceptible to xenotransplantation. Hence, they are suitable to be used to establish a model for human tumour xenotransplantation. Our results showed that as low as 100 BCSCs were able to form tumours in NOD/ SCID mice within 30 days, with a tumour formation rate of 91.7%; whilst a 100‐fold increased MCF‐7 cells (1 × 10^4^) failed to form tumours in mice. The tumour formation rate in mice reached 100% with only 500 BCSCs within 18 days, which was far more efficient than that with 1 × 10^5^ MCF‐7 cells. Taken together, our tumorigenesis experiment results showed that BCSC exhibited a very strong tumorigenic ability, which was significantly different from that of MCF‐7.

As the source of tumour occurrence, development, recurrence and metastasis, CSCs are resistant to tumour treatment methods due to their high degree of self‐renewal and differentiation ability.^28^, ^29^, ^30^, ^31^ BCSCs in breast cancer contribute to malignant progression; hence, targeting BCSC may fundamentally suppress cancer and improve therapeutic efficacy.^32^, ^33^, ^34^, ^35^, ^36^, ^37^, ^38^ Our analysis of cell proliferation using CCK‐8 showed that the cell killing capacity of Ad‐VT, Ad‐T and Ad‐VP3 on BCSC increased in a dose‐dependent manner. At the same dosage, the cell killing capacity increased over time, with the highest rate observed at 96 hours, followed by 72, 48 and 24 hours (*P* < 0.05). Notably, at 96 hours, the Ad‐VT at 100 MOI exhibited the strongest cell killing ability on BCSCs at about 82%. Our results showed that Ad‐VT had a significant cell killing effect on BCSCs.

Flow cytometry analysis demonstrated that, in BCSCs infected with Ad‐VT at 100 MOI and with Ad‐VT at 10 MOI, the proportion of CD44^+^CD24^−^ cell subsets decreased significantly in a dosage‐ and time‐dependent manner, which was significantly different from that of the control group (*P* < 0.05). The inhibitory effect of Ad‐VT at 100 MOI on CD44^+^CD24^−^ cells was significantly stronger than that of Ad‐VT at 10 MOI (*P* < 0.05). Our results showed that Ad‐VT can reduce the proportion of CD44^+^CD24^−^ cells in BCSCs and can fundamentally kill BCSCs.

When cells undergo apoptosis, the biological morphology of the cell membrane and nucleus changes, including eversion of membrane phospholipids, and break down of nuclear nucleolus. Annexin is a calcium ion‐dependent phospholipid‐binding protein that can specifically bind phospholipid serine (PS) inside the phospholipid bilayer of cells. When BCSCs undergo early apoptosis, the PS inside the everted cell membrane specifically binds Annexin V, which can then be detected by FITC green fluorescence. As the cell membrane remains intact, PI is inaccessible to the cell to stain the nucleus. However, at later stages of apoptosis, whilst the damaged cell membrane can be dyed green by FITC due to Annexin V binding, the nucleus can be stained red by PI. Although necrotic cells have no membrane phospholipid eversion, an increased cell membrane permeability enables PI to enter the cell to stain the nucleus. Accordingly, BCSCs without undergoing apoptosis show no staining.

Under fluorescence microscope, BCSC apoptosis was observed after infection with Ad‐VT, Ad‐T and Ad‐VP3. The cell membrane of apoptotic cells was stained green, whilst the nucleus was stained red with nuclear fragmentation. Consistently, flow cytometry analysis showed that Ad‐VT exhibited the strongest ability to induce apoptosis, followed by Ad‐T and Ad‐VP3. The apoptosis rates were observed to be increased in a time‐dependent manner. Taken together, our results indicate that Ad‐VT mainly kills BCSC through apoptosis.

Next, we analysed changes in the mitochondrial membrane potential (MMP) using fluorescent probe JC‐1. In normal cells, the high MMP enables the JC‐1 to enter the mitochondrial matrix where a polymer is formed to emit red signal at a high concentration. Conversely, when the cell undergoes apoptosis, decreased MMP prohibits the JC‐1 from entering the mitochondria to form a polymer, so JCI remains in the cytoplasm as a monomer that emits green signal. Accordingly, the apoptosis can be detected by observing the fluorescent colour of JC‐1.

After 72 hours of infection with recombinant adenovirus, normal cells showed red signals, whilst apoptotic cells showed green signals following JC‐1 staining. Ad‐VT, Ad‐T and Ad‐VP3 were able to induce apoptosis of BCSCs by decreasing the MMP of apoptotic cells to be stained green by JC‐1. Compared to cells infected by Ad‐T and Ad‐VP3, a higher rate of apoptosis was observed in cells infected by Ad‐VT. In agreement with the results obtained by JC‐1 staining, quantitative analysis of MMP changes by TMRM staining showed that MMP decreased most significantly in Ad‐VT infected cells.

In summary, we successfully isolated and cultured breast cancer stem cells, and found that recombinant adenovirus Ad‐VT had a killing effect on BCSCs, and that the killing effect on BCSCs was mainly caused by apoptosis. Our results provide a new theoretical basis for the future treatment of breast cancer.

## CONFLICT OF INTERESTS

The research was conducted in the absence of any commercial or financial relationships that could be deemed as a potential conflict of interest.

## AUTHOR CONTRIBUTION

**Wenjie Li:** Conceptualization (equal); Data curation (equal); Formal analysis (lead); Methodology (equal); Software (equal); Writing‐original draft (equal); Writing‐review & editing (equal). **Yiquan Li:** Conceptualization (equal); Data curation (equal); Formal analysis (lead); Methodology (equal); Software (equal); Writing‐original draft (equal). **Yingli Cui:** Conceptualization (equal); Data curation (equal); Formal analysis (equal); Methodology (equal). **Shanzhi Li:** Conceptualization (equal); Formal analysis (equal); Methodology (equal). **Yilong Zhu:** Conceptualization (equal); Methodology (equal). **Chao Shang:** Conceptualization (equal); Methodology (equal). **Gaojie Song:** Conceptualization (equal); Methodology (equal). **Zirui Liu:** Conceptualization (equal); Methodology (equal). **Zhiru Xiu:** Conceptualization (equal); Methodology (equal). **Jianan Cong:** Conceptualization (equal); Methodology (equal). **Tingyu Li:** Conceptualization (equal); Methodology (equal). **Xiao Li:** Conceptualization (equal); Formal analysis (equal); Funding acquisition (equal); Investigation (equal); Methodology (equal); Project administration (lead); Writing‐review & editing (equal). **Lili Sun:** Conceptualization (equal); Data curation (equal); Formal analysis (equal); Methodology (equal); Project administration (equal). **Ningyi Jin:** Conceptualization (equal); Data curation (equal); Funding acquisition (lead); Methodology (equal); Writing‐review & editing (equal).

## ETHICAL APPROVAL

All applicable international, national and/or institutional guidelines for the care and use of animals were followed.

## Data Availability

The data that support the findings of this study are available from the corresponding author upon reasonable request.
